# Secreted Factors from Keloid Keratinocytes Modulate Collagen Deposition by Fibroblasts from Normal and Fibrotic Tissue: A Pilot Study

**DOI:** 10.3390/biomedicines8070200

**Published:** 2020-07-08

**Authors:** Mansour A. Alghamdi, Laith N. AL-Eitan, Andrew Stevenson, Nutan Chaudhari, Nicole Hortin, Hilary J. Wallace, Patricia L. Danielsen, Mitali Manzur, Fiona M. Wood, Mark W. Fear

**Affiliations:** 1 Department of Anatomy, College of Medicine, King Khalid University, Abha 61421, Saudi Arabia; m.alghamdi@kku.edu.sa; 2 Genomics and Personalized Medicine Unit, College of Medicine, King Khalid University, Abha 61421, Saudi Arabia; 3 Department of Applied Biological Sciences, Jordan University of Science and Technology, Irbid 22110, Jordan; lneitan@just.edu.jo; 4 Department of Biotechnology and Genetic Engineering, Jordan University of Science and Technology, Irbid 22110, Jordan; 5 Burn Injury Research Unit, School of Biomedical Sciences, Faculty of Health and Medical Sciences, The University of Western Australia, 35 Stirling Highway, Crawley 6009, Australia; andrew@fionawoodfoundation.com (A.S.); 22021115@student.uwa.edu.au (N.C.); nicole.hortin@uwa.edu.au (N.H.); hilary.wallace@nd.edu.au (H.J.W.); fiona.wood@health.wa.gov.au (F.M.W.); 6 School of Medicine, The University of Notre Dame Australia, Fremantle 6959, Australia; 7 Department of Dermatology and Copenhagen Wound Healing Center, Copenhagen University Hospital, DK-2400 Copenhagen NV, Denmark; patriciadanielsen@yahoo.dk; 8 Telethon Kids Institute, Perth Children’s Hospital, The University of Western Australia, Nedlands 6009, Australia; mitali.manzur@telethonkids.org.au; 9 Burns Service of Western Australia, Perth Children’s Hospital and Fiona Stanley Hospital, Department of Health, Perth 6009, Australia; 10 Fiona Wood Foundation, Fiona Stanley Hospital, Murdoch, Perth 6150, Australia

**Keywords:** keloids, keratinocytes, collagen type I, wound healing, fibroblasts, coculture techniques

## Abstract

Interactions between keratinocytes and fibroblasts in the skin layers are crucial in normal tissue development, wound healing, and scarring. This study has investigated the role of keloid keratinocytes in regulating collagen production by primary fibroblasts in vitro. Keloid cells were obtained from removed patients’ tissue whereas normal skin cells were discarded tissue obtained from elective surgery procedures. Fibroblasts and keratinocytes were isolated, cultured, and a transwell co-culture system were used to investigate the effect of keratinocytes on collagen production using a ‘scar-in-a-jar’ model. Keloid fibroblasts produced significantly more collagen than normal skin fibroblasts in monoculture at the RNA, secreted protein, and stable fibrillar protein level. When keloid keratinocytes were added to normal skin fibroblasts, expression of collagen was significantly upregulated in most samples, but when added to keloid fibroblasts, collagen I production was significantly reduced. Interestingly, keloid keratinocytes appear to decrease collagen production by keloid fibroblasts. This suggests that signaling in both keratinocytes and fibroblasts is disrupted in keloid pathology.

## 1. Introduction

Keloid scarring is an abnormal fibroproliferative process that can occur as a result of skin trauma. Keloid scar is characterized by progressive and excessive accumulation of disordered collagen types I and III during wound repair [1,2]. The molecular mechanisms that regulate fibroblast proliferation and collagen metabolism in keloids are poorly understood. A significant increase in type I procollagen-specific mRNA levels and type I/III procollagen mRNA ratio is observed in keloid fibroblasts (KFs) compared with normal skin fibroblasts (NFs) [3,4]. Others have found higher levels of collagen type I and III (protein and mRNA) produced by KFs isolated from the growing margin compared with extralesional and intralesional sites [5], suggesting an association between the continuous growth at the wound boundary and collagen production.

Communication between keratinocytes and fibroblasts is crucial in wound healing. It has been shown that normal keratinocytes (NKs) tend to have enhanced proliferation, migration, and morphology in the presence of NFs, favoring co-cultured in direct contact with NFs than in the transwell [6,7]. In keloids, NFs proliferate more when co-cultured with NKs or keloid keratinocytes (KKs) compared to culturing in the absence of keratinocytes, significantly higher in the presence of KKs rather than NKs [8]. Interestingly, KFs was of a significant proliferation when co-cultured with KKs compared to NKs co-culture [8,9,10].

The critical role of epithelial-mesenchymal interactions has been linked to skin regeneration, wound healing, and scarring [6,7,9,11,12,13,14,15,16,17]. Several experimental models have been employed to study epithelial-mesenchymal interaction in keloid scars. These include organotypic skin constructs, the use of conditioned media, and indirect contact using transwell membrane inserts [12,16,17,18]. A significant contraction has been shown in murine type I collagen gels populated with NFs upon keratinocytes adding to the gel compared to the control group (no keratinocytes) [19]. Moreover, a greater contraction was observed in collagen lattice populated with KFs compared to NFs populated lattices. Similarly, an organotypic skin construct cultured with KFs/NKs showed increased contracture with more organized α-smooth muscle actin in the dermal layer compared to the NFs/NKs construct [17].

Indeed, epithelial-mesenchymal interaction may have a regulatory role in collagen synthesis with the presence of keratinocytes influencing collagen production by dermal fibroblasts [16,17,18]. A reduction in type I collagen mRNA (COL1A1) level was observed in the treatment of NFs with NKs conditioned medium which is not seen with the fibroblast conditioned medium [12]. Another study found no significant effect on COL1A production when NKs added to NFs [9]. In contrast, an increased level of [H3] proline incorporation in the media collected from co-culturing NFs with KKs and NKs has been observed compared to control (NFs without keratinocytes) [18] suggesting increased collagen production. Another study found an increase in soluble collagen I and III productions by NFs and KFs when co-cultured with keratinocytes, especially KKs [16].

These studies used different experimental models to study epithelial-mesenchymal interaction in keloid scars in relation to collagen production. The contradictory results reported in previous studies can be explained in one hand by the variation in experimental models used and on the other hand by the quantitative method used for collagen detection which may require for example destroying of cell layers when counting cell numbers or measuring proteins [16,18]. In contrast to these other models, the “scar-in-a-jar” model addresses many of these limitations by allowing quantitation of collagen without destroying cell layers, visualization of collagen structure, quantitation of cell numbers, the use of co-factors such as ascorbic acid, a short culture time and direct quantification of specific proteins in a single-well format [20]. In this study, we have used a ‘scar-in-a-jar’ model, together with transwell co-culture, to measure the fibrillar COL1A deposition by NFs and KFs in the presence of normal/keloid keratinocytes.

## 2. Materials and Methods

### 2.1. Subjects

Keloid tissue and normal skin were used following ethical approval from the University of Western Australia and Royal Perth Hospital, Perth, WA, Australia in accordance with the Declaration of Helsinki 1975, as revised in 2013 with written informed consent obtained from all participants. Surgically excised keloid scars were collected from six patients (Table 1). Samples were collected, isolated, and frozen in 2013 until being used in this study between 2015–2016. Patients had received no previous treatment of the keloid scar before surgical excision. A full history was taken prior to excision and clinical examination was performed by a plastic surgeon to confirm the diagnosis. Clinical criteria used to differentiate keloid from normotrophic scar include a history of continuous growth outside the boundaries of the wound and symptoms such as pain and itch. KFs and KKs were isolated from all keloid samples (KF *n* = 6; KK *n* = 6). Control fibroblasts and keratinocytes were isolated from normal skin tissue obtained from discarded tissue after elective surgery. NF1 and NK1 were isolated from the inner upper arm skin of a subject who underwent a skin reduction surgical procedure secondary to major weight loss. NF2 were also isolated from the forearm skin of an additional healthy individual.

### 2.2. Isolation and Culture of Fibroblasts

The surgically excised keloid tissues were collected in Dulbecco’s Modified Eagle’s Medium (DMEM): Nutrient Mixture F-12 (DMEM/F12, GIBCO^®^, Carlsbad, CA, USA) and processed within 4 h. Fibroblasts were isolated from the center of fresh keloid tissue by explant method, as described previously by Keira et al. and Tucci-Viegas et al. with slight modification [21,22]. Briefly, the isolated dermis was fragmented into 5–10 mm^2^ in size fragments that transferred to petri dish containing DMEM with 10% fetal bovine serum (FBS, Invitro technologies, Noble Park North, Australia) and 1% Penicillin/Streptomycin (P/S) and incubated at 37 °C in 5% carbon dioxide (CO_2_) atmosphere. When cells reached 80% confluence, the media and the fragments were discarded, and cells were washed with PBS, trypsinized with 0.05% Trypsin-EDTA (GIBCO^®^, Life Technologies, Grand Island, NY, USA). After all, cells were placed in a media containing 10% FBS to inactivate the trypsin to be seeded into a T75 flask. Cells growth was maintained until the second passage when cells were resuspended in freezing medium of DMEM containing 10% dimethylsulfoxide (DMSO) and kept in liquid nitrogen for further use.

### 2.3. Isolation and Culture of Keratinocytes

Keloid tissues were surgically excised, collected and processed in DMEM within 4 h. Keratinocytes were isolated from fresh keloid tissue by the digestion method using a modified version of the previously published protocol [23,24]. Briefly, samples were placed in keratinocytes growth media (KGM, EpigrowTM, human epidermal keratinocyte complete culture media kit, Merck, Millipore, MA, USA) where the tissue was cut into strips of 3–4 mm^2^ to allow dispase to infiltrate the tissue. Tissue strips were incubated overnight at 4 °C in 5 mL KGM containing 0.0127 g of dispase powder, 20 μL Fungizone, and 100 μL kanamycin. The next day, the separated epidermis was spread out flat on the surface of the TrypLE drop with the basal layer downward and incubated for 20 to 30 min at room temperature. The epidermis was rubbed to generate a single cell suspension that washed with KGM to concentrate the cells down to the bottom of the dish. After centrifuging single cells suspension, the cell pellet was resuspended in KGM with 1% P/S and seeded in a T25 flask (25 cm^2^, Greiner Bio-One, Germany). Cell growth was maintained until the second passage, and cells were kept frozen in liquid nitrogen until being used.

### 2.4. qPCR for COL1A

RNA and cDNA were prepared from lysed cells cultured for 24 h, using Qiagen RNeasy Mini Kit and QuantiTect Reverse Transcriptase Kit (QIAGEN, Valencia, CA, USA), following manufacturer’s instructions. Quantitative PCR was then performed using QuantiTect SYBR Green PCR kit and QuantiTect primers COL1A1 (QIAGEN, Valencia, CA, USA), housekeeping primers GAPDH and ACTB or PGK. qPCR was performed on Step One System machine from ThermoFisher following optimal thermal cycles as recommended by Qiagen for SYBR Green products. Relative expression of Col1 was calculated following the Pfaffl method with multiple reference genes, taking into consideration primer efficiency differences of no more than 6%. Each sample was compared to the mean Ct value of all normal (non-keloid) cell lines at each respective time point. Results are expressed as a fold change.

### 2.5. Fluorescent Resonance Energy Transfer (FRET) Assay to Quantitate Procollagen I

A Fluorescence Resonance Energy Transfer (FRET) assay was carried out to measure procollagen concentration in cell media using the human pro-collagen type 1 kit from Cisbio (Cisbio Inc., Bedford, MA), according to manufacturer’s instructions. Then 16 μL of cell media was added to a single well of the 384 well plate, (Greiner 384 low volume white plates, high base 4–25 μL working volume), to which 2 μL of the anti-human procollagen cryptate antibody and 2 μL of the anti-human procollagen d2 antibody was also added. A standard curve was made up the same way, with the procollagen standards ranging from 0.78–100 ng/mL, as well as a negative control and a cryptate control. Each sample was run in triplicate. The plate was incubated at room temperature overnight, then read on a BMG Clariostar microplate reader. Microplate reader settings were as follows: excitation filter—330 nm, emission filters—620 nm and 665 nm, integration delay (lag time) 60 μs, integration time 400 μs, number of flashes—200, gain—2400. For each well, the ratio of the 665 nm/620 nm was calculated, then the mean ratio calculated using the triplicate wells for each sample and standard. The delta F% was then calculated by using the following equation—(ratio of the standard or sample—ratio negative control)/ratio negative control × 100. The delta F% values for the standard curve were then plotted, the equation of the line worked out and the procollagen concentration of each sample calculated.

### 2.6. Fibroblast and Keratinocyte Co-Culture

**Figure 1:**
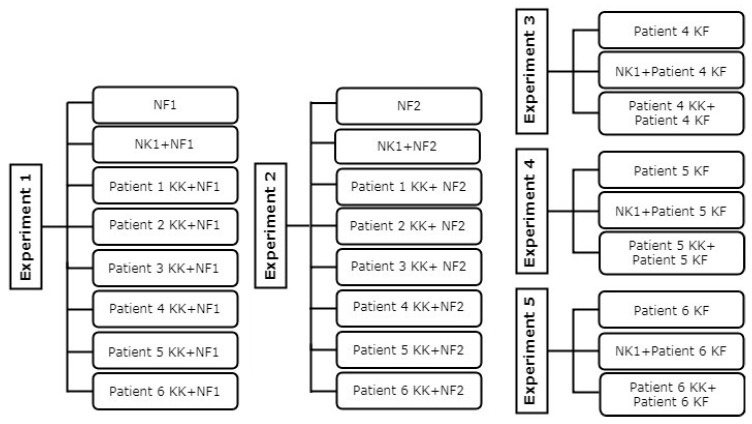
Flowchart of the experimental design. Normal fibroblast (NF), normal keratinocyte (NK), keloid fibroblast (KF), keloid keratinocyte (KK).

A total of six KFs, six KKs, two NFs, and one NK were used in this study (Figure 1). The co-culture experiments were carried out as outlined earlier with some modifications [25]. KFs and NFs were seeded at a density of 5 × 10^4^ cells/well in 24-well plate in DMEM with 10% FBS and 1% P/S. KKs and NKs were seeded at a density of 1 × 10^5^ cells on transwell membrane inserts in a 24-well plate (Corning incorporated, Costar, USA) in KGM with 1% P/S or in DMEM with 10% FBS and 1% P/S (as control experiment). After 24 h incubation at 37 °C in 5% CO_2_, KFs and NFs media were removed and replaced by un-stimulated media (DMEM with 1% P/S, 0.5% FBS and 1% of 100 mM L-ascorbic acid 2-phosphate (Sigma Aldrich, Saint Louis, MO, USA)). The cultured KKs and NKs on the transwell membrane inserts were washed with PBS and transferred onto the cultured KFs and NFs in a 24-well plate containing un-stimulated media. Each co-culture condition was performed in duplicate with the experiment maintained at 37 °C in 5% CO_2_ for 6 days before staining.

### 2.7. Staining for Collagen Type I

The staining for COL1A was carried out using the ‘scar-in-a-jar’ model developed by Chen et al. with some modifications [20]. The cultured fibroblasts were blocked with 3% bovine serum albumin (BSA, Sigma-Aldrich, St. Louis, MO, USA) in FluoroBrite DMEM (GIBCO^®^, Life Technologies, Grand Island, New York, NY, USA) for 30 min at room temperature. Primary antibody (10AB, monoclonal mouse anti-human collagen type I, Santa Cruz Biotechnology, Dallas, TX, USA) was added to the cells and incubated at 37 °C in 5% CO_2_ for 90 min followed by one time washing with FluoroBrite DMEM. A 4% paraformaldehyde (Sigma Aldrich, Saint Louis, MO, USA) in PBS was added to the cells and incubated for 10 min at room temperature and washed two times with FluoroBrite DMEM. Secondary antibodies (AlexaFluor 488 goat anti-mouse IgG, Life Technologies, Eugene, Oregon, OR, USA) was added and incubated for 30 min followed by a one-time wash with FluoroBrite DMEM. The nucleus was stained for 10 min at room temperature with Hoechst (Cat. No. H3570, Life Technologies, Carlsbad, CA, USA) and washed two times with PBS, leaving the last wash in the wells for imaging.

### 2.8. Blind Assessment

The image assessor was blinded to the identity of all experimental conditions to exclude any potential bias when assessing the collagen quantitation. After analysis completion, the experimental images were unblinded.

### 2.9. Whole Well Imaging for Collagen Quantitation

**Figure 2:**
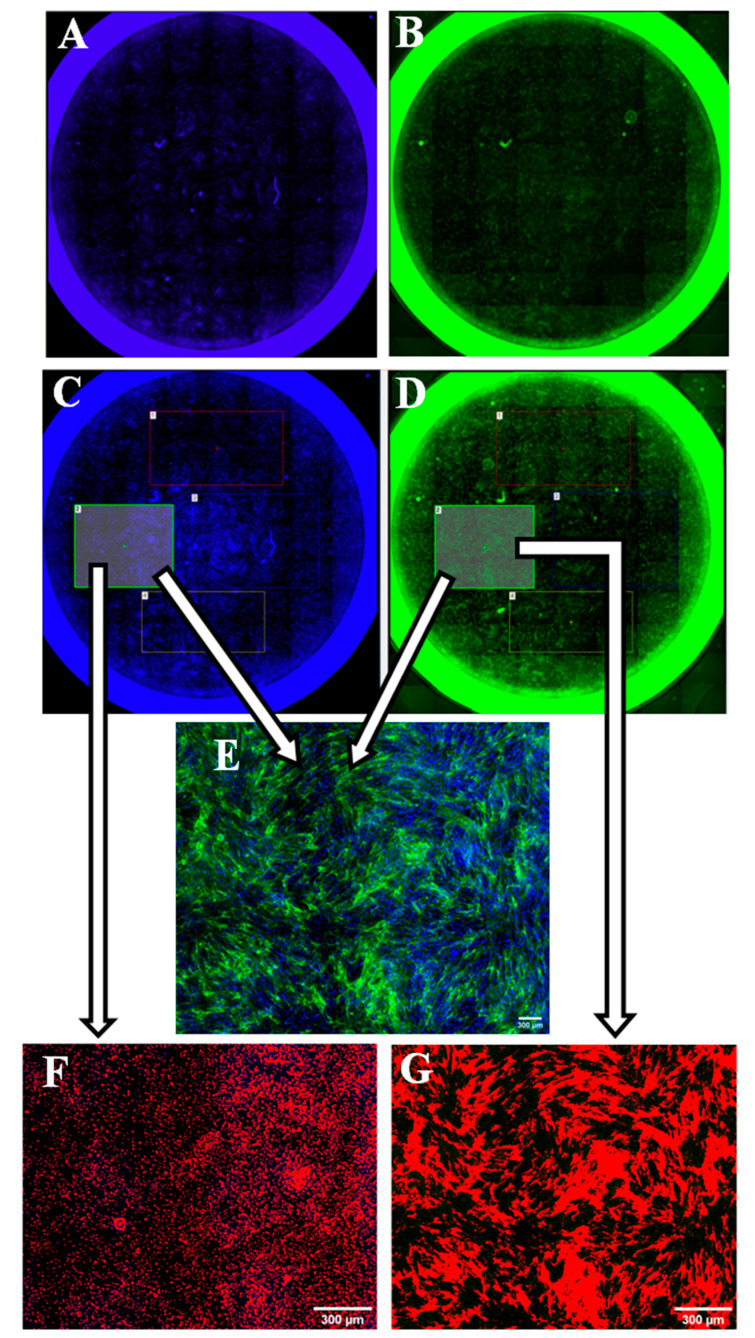
Whole well imaging and collagen quantification process. The left column shows the process of counting cell nuclei (whole well imaging (**A**), regions of interest (ROI) selection (**C**), and nuclei count (**F**)). The right column shows the process of measuring the collagen binary area (whole well imaging (**B**), ROI selection (**D**), and collagen threshold (**G**)). (**E**): collagens and nuclei merged.

Imaging of the co-culture was undertaken using Nikon inverted research microscope (TE 300) and Nikon NIS-Elements software, Version: Ver.30.01 DU1, (Nikon, Japan). The entire well was imaged by a tile scan using a 4X objective, B2-A (488 nm) and DAPI (358 nm) filter blocks. The same imaging alignments were used for both the collagen and nuclear staining (Figure 2). The exposure time was set at 300 ms for the Hoechst stain and 1 s for the collagen staining (488 nm excitation). For each well, a number of ‘regions of interest’ (ROIs) (~3–6 squares) were used. Each square area had a binary threshold applied to mark either the cell nuclei or the collagen fibers. Once the threshold was adjusted, the object counts, and binary area covered were measured (Figure 2). For each well, the sum of binary areas obtained from measuring the collagen staining was divided by the sum of its corresponding object count obtained from measuring the nuclear staining to give an estimation of the amount of collagen secreted by each cell.

### 2.10. Statistical Analysis

Statistical comparison was carried out using the obtained ROI from collagen quantitation. Statistical significance between selected groups was calculated using the Mann-Whitney *U*-test (nonparametric) with *p*-value < 0.05 was considered significant. All statistical analyses were done using Prism software v.6.0 (GraphPad, La Jolla, CA, USA).

## 3. Results

### 3.1. Collagen Production of Keloid Fibroblasts Is Significantly Higher Than Normal Skin Fibroblasts in Monoculture

**Figure 3:**
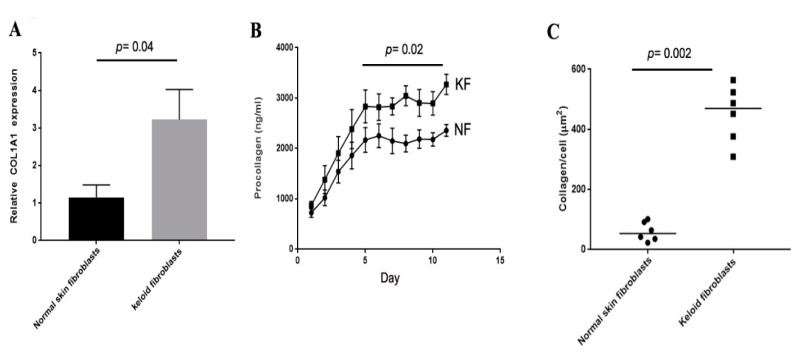
Collagen production of keloid fibroblasts is significantly higher than normal skin fibroblasts in monoculture. qPCR for COIA1 shows higher levels in keloid fibroblasts compared to normal skin fibroblasts at 24 h after plating in culture (**A**). Fluorescent Resonance Energy Transfer (FRET) assay shows increased levels of procollagen peptide in cell media over a period of 11 d from keloid fibroblasts (KF) compared to normal skin fibroblasts (NF) (**B**). Immunohistochemistry for COLIA1 using the ‘scar-in-a-jar’ model shows significantly higher collagen I deposited by keloid fibroblasts compared to normal skin fibroblasts (**C**).

Collagen I production was compared in the keloid patient and normal skin fibroblast samples in monoculture. Twenty-four hours after plating RNA was isolated and COL1A levels were assessed by qPCR. Keloid fibroblasts expressed significantly higher levels of COL1A than normal skin fibroblasts (Figure 3A, *p* = 0.04). QPCR at later timepoints showed no sustained difference in COLIA1 RNA levels (data not shown). Secreted protein levels were measured using a Fluorescent Resonance Energy Transfer (FRET) assay and showed significantly higher levels of collagen I in KFs compared to control (Figure 3B). Finally, immunohistochemistry of deposited collagen I showed significantly higher fibrillar collagen I in keloid fibroblasts (Figure 3C) when measured at 6 d using the ‘scar-in-a-jar’ model.

### 3.2. Keloid Keratinocytes Significantly Increase Collagen Production by Normal Skin Fibroblasts

**Figure 4:**
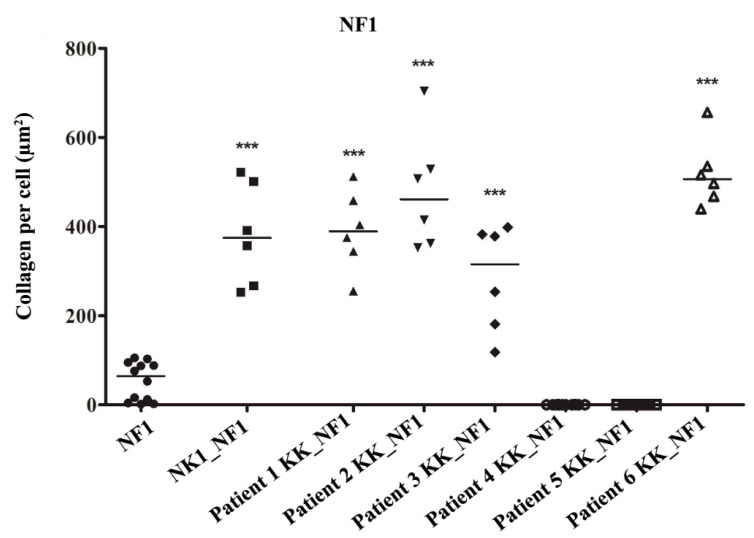
Keloid keratinocytes significantly increase collagen production by normal skin fibroblasts. Keloid keratinocytes and normal skin keratinocytes were co-cultured with normal fibroblasts from patient 1 (NF1). Both normal keratinocytes and 4/6 keloid keratinocyte samples significantly increased COLI produced by NF1. *** = *p*-value < 0.001.

**Figure 5:**
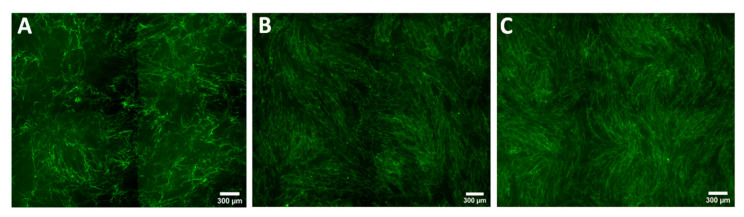
Comparison of the COL1A production evaluated by immunohistochemistry staining of normal fibroblasts culture from patient 1 (NF1) (**A**), when co-cultured with normal keratinocytes (NK1) (**B**), or keloid keratinocytes (Patient 1 keloid keratinocytes (KK)) (**C**).

COL1 production was significantly increased by normal fibroblasts from patient 1 (NF1) when co-cultured with patients 1, 2, 3, and 6 KK (*p* < 0.001) compared with NF1 cultured alone (Figure 4 and Figure 5). The addition of KKs from patients 4 and 5 led to a significant decrease in COL1 production by NF1 (*p* < 0.001). When NF1 was cocultured with NK1 there was also a significant increase in COLI (*p* < 0.001) (Figure 5). No significant difference between the effects of NK1 and KK co-culture was observed.

**Figure 6:**
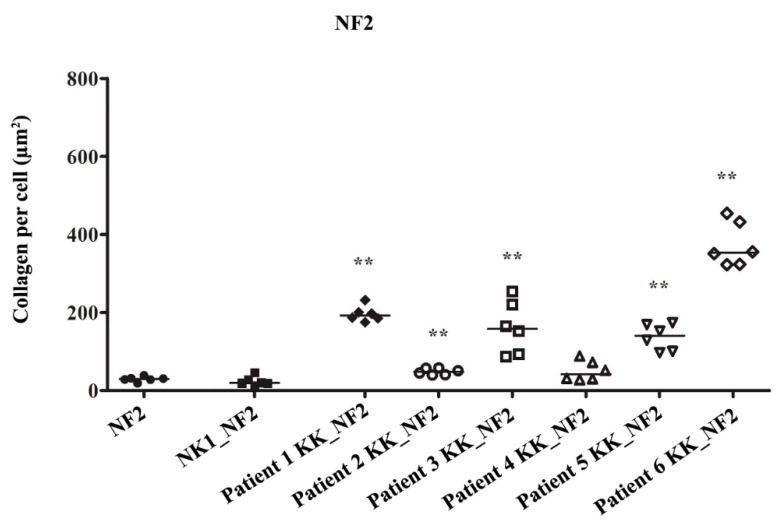
Keloid keratinocytes significantly increase collagen production by normal skin fibroblasts. Keloid keratinocytes and normal skin keratinocytes were co-cultured with normal fibroblasts from patient 2 (NF2). 5/6 keloid keratinocyte samples increased NF2 COLI production. No effect was seen with normal keratinocytes or with 1 keloid keratinocyte sample. ** = *p*-value < 0.01.

**Figure 7:**
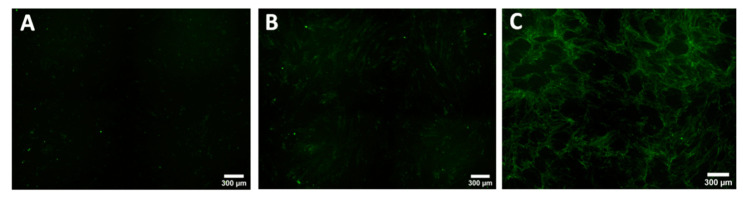
Comparison of the COL1A production evaluated by immunohistochemistry staining of normal fibroblasts culture from patient 2 (NF2) (**A**), when co-cultured with normal keratinocytes (NK1) (**B**), or keloid keratinocytes (Patient 1 keloid keratinocytes (KK)) (**C**).

In normal fibroblasts from patient 2 (NF2) the addition of NK1, did not increase collagen production (*p* = 0.31). The amount of collagen produced by NF2 increased significantly by variable amounts when treated with KKs from patients 1, 2, 3, 5, and 6 (*p* = 0.002) compared to being cultured alone (Figure 6 and Figure 7). KKs from patient 4 did not increase collagen production by NF2 (*p* = 0.24) (Figure 6).

### 3.3. Collagen Production by Keloid Fibroblasts Is Reduced in the Presence of Keloid Keratinocytes

**Figure 8:**
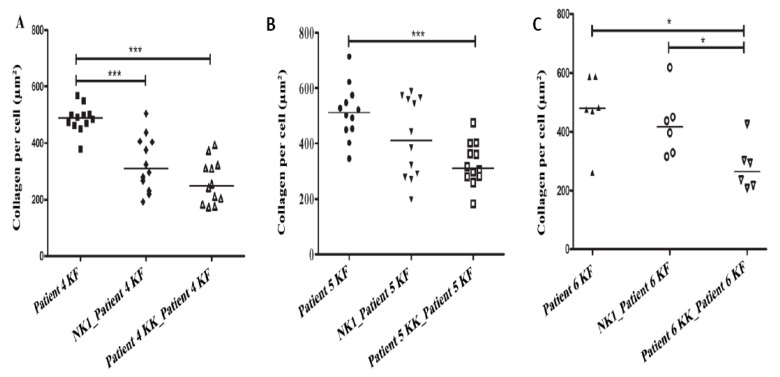
Keloid keratinocytes significantly decrease collagen production by keloid fibroblasts. Keloid fibroblasts from patients 4, 5 and 6 were cultured with matched keloid keratinocytes or normal keratinocytes (NK1). Keloid keratinocytes significantly reduced COLI produced by the matched keloid fibroblasts (**A**–**C**). * = *p*-value < 0.05, *** = *p*-value < 0.001.

**Figure 9:**
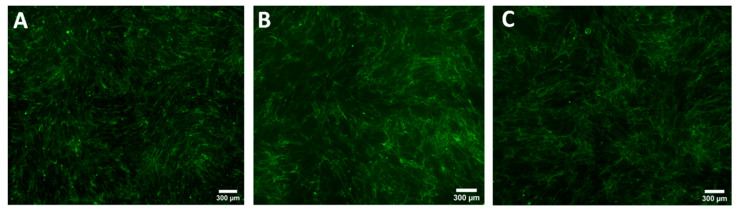
Comparison of the COL1A production evaluated by immunohistochemistry staining of keloid fibroblasts culture from patient 4 (KF) (**A**), when co-cultured with normal keratinocytes (NK1) (**B**), or keloid keratinocytes (Patient 4 keloid keratinocytes (KK)) (**C**).

A significant reduction in COL1A by KFs was found when they were co-cultured with matched KKs in patients 4 and 5 (*p* < 0.001) and patient 6 (*p* = 0.015) (Figure 8A–C). A reduction in COL1A production by KFs was also observed when non-matched NK1 were added; however, this was only significant in patient 4 (*p* < 0.001) (Figure 9). In patient 6 only the amount of collagen was significantly less after adding KKs compared with NK1 co-culture (*p* = 0.015) (Figure 8C). For patients no. 1, 2, and 3, there were not enough KFs cells to co-culture with their corresponding KKs cells.

## 4. Discussion

This study used the “scar-in-a-jar” model to investigate the role of keratinocytes in controlling collagen production by fibroblasts. In monoculture keloid fibroblasts produced significantly more collagen than normal skin fibroblasts. Keloid keratinocyte co-culture was shown to increase normal skin fibroblast collagen deposition whilst reducing keloid fibroblast collagen deposition. However, not all keloid keratinocyte samples had this effect, wherein four out of the six keloid keratinocytes increased collagen synthesis and deposition by normal fibroblasts, but the other two keloid keratinocytes had the opposite effect. Furthermore, they significantly decreased collagen synthesis and deposition by normal fibroblasts, suggesting significant intraindividual variability. Interestingly, the addition of normal skin keratinocytes also appeared to reduce collagen produced by keloid fibroblasts, although not to the same extent as keloid keratinocytes.

The overall increase in collagen production by NFs when co-cultured with keloid keratinocytes observed in this study correlates with several previous studies [16,18]. In contrast, a reduction in COLIA1 synthesis has been reported in the co-culture of NFs with NKs using culture plate inserts or by adding keratinocyte conditioned media [11,12]. Bellemare et al. found that the addition of NKs to dermal sheets (reconstructed using NFs isolated from normal human skin) had no significant effect on the production of collagen type I [9]. However, in this study the addition of NK1 increased collagen production in NF1 but not NF2. One explanation for this may be that NK1 and NF1 were isolated from the same skin, and the variable response observed may reflect the variations reported in previous studies [9,11,12]. KKs from two patients decreased the production of COL1A significantly by NF1 but not NF2. The ability of normal fibroblasts to respond to the addition of KKs can be affected by several elements such as differences between keloid samples (e.g., severity of the disease), differences between normal skin samples, body site, and age of individuals. For example, the level of collagen deposition has shown to be reduced as the age of tissues and cells advanced [26]. In terms of body site, NF1 was isolated from the upper arm which is a region known to develop keloid scar while NF2 was isolated from the forearm, a less prone area to keloid formation [2]. Broadly, these results are important to consider in therapeutic interventions since it appears not all patient cells are expressing the same signaling molecules or have the same phenotype, despite all being from keloid scars. Understanding the molecular basis for the heterogeneity of the responses identified here will be an important consideration for future intervention into keloid scars. Further work examining the profile of secreted molecules, potentially using a proteomic approach, may help identify key signaling components for future study.

Our findings showed that the treatment of KFs with KKs led to a marked reduction in collagen production in all of the three patient samples tested. There was a similar trend observed when NKs were added but was only significant in one patient. The contradictory results reported in the current study compared to previous studies may result from several issues including sample demographic data and methodology. There are several technical differences between studies, including lower cell density in this study and the use of the ‘scar-in-a-jar’ method, both of which can impact on collagen production [27,28]. In this study, samples were also collected from different anatomical body sites (e.g., chest, neck, shoulder, and sternum) while control samples were from the upper arm which is an area known to develop keloid scars. Lim et al. and Phan et al. used samples collected only from the earlobe and for their control used samples from foreskin [16,18]. The use of primary adult cells and multiple samples from different patients supports that the current findings likely reflect the diversity and nature of the response. Another difference that may contribute is the quantitative method used. This study quantifies collagen production via direct staining of deposited collagen without the need of destroying ECM or cells. In contrast, previous studies have used RNA and protein lysates to perform western blot and northern blot which requires destruction of cell layers in order to quantify collagen [16,18].

Altered collagen production by fibroblasts in coculture with keloid keratinocytes may be linked to signals required for wound closure [29]. The effects may be mediated by cytokines such as IL-1 and IL6 [29,30], or to secreted microvesicles or exosomes that are increasingly being identified as important in epithelial-mesenchymal communication [31,32]. Overall, the results of this study suggest that the synthesis of COL1A in fibroblasts is at least partly regulated by soluble factors released by keratinocytes and that these pathways are altered in keloid disease. This suggests that keratinocyte:fibroblast cross-talk may be a potential avenue for future therapies. Further exploration is needed to clarify the role of epithelial:mesenchymal signaling in keloid pathogenesis. Moreover, the significant variation observed likely reflects true biological variation observed clinically and may be a reason for previous conflicting reports. Here, the use of primary cells from multiple adult patients and body sites provides additional insight above that of many models previously used into the nature of the keratinocyte–fibroblast interaction in keloid disease. The main limitation of this study is the use of keloid tissue with differences in severity of disease, body sites, age, and ethnicity of individual. Future work taking these factors into consideration is needed to better understand the changes in biology associated with keloid scarring. Another limitation in this study includes the use of small sample size.

## 5. Conclusions

Our findings revealed that keloid keratinocyte modulation of collagen production by both keloid and normal fibroblasts suggests important signaling between these cells in keloid disease. The difference in response between normal and keloid fibroblasts also suggests significant dysregulation of normal signaling pathways. Further work to identify the key factors and dysregulated pathways may provide an opportunity for future therapeutic amelioration of keloid scars.

## Figures and Tables

**Table 1:** Participated subjects’ data.

Patient ID.	Age	Gender ^1^	Phenotype	Site of Excision or Biopsy	Ethnicity
			(Scare Age/Year)		
**Patient 1**	53	M	Keloid (25)	Sterunm	Northwest European
**Patient 2**	42	M	Keloid (5)	Ear	Northwest European
**Patient 3**	30	M	Keloid (15)	Sterunm	East Asian
**Patient 4**	27	F	Keloid (10)	Shoulders	East Asian
**Patient 5**	47	F	Keloid (3)	Sterunm	Northwest European
**Patient 6**	29	M	Keloid (6)	Sterunm	East Asian
**Control subject 1**	35	F	Normal	Inner upper arm	Unknown
**Control subject 2**	25	M	Normal	Forearm	European

^1^ Male (M), Female (F).

## Abbreviations

KFs	Keloid fibroblasts
KKs	keloid keratinocytes
NFs	Normal fibroblasts
NKs	Normal keratinocytes
KGF	Keratinocyte growth factor
COL1A	Collagen type I
KF	Keloid fibroblasts
KK	Keloid keratinocytes
P/S	Penicillin/Streptomycin
DMSO	Dimethylsulfoxide
DMEM	Dulbecco’s Modified Eagle’s Medium
KGM	keratinocytes growth media
